# The Latex Protein MLX56 from Mulberry (*Morus multicaulis*) Protects Plants against Insect Pests and Pathogens

**DOI:** 10.3389/fpls.2017.01475

**Published:** 2017-08-23

**Authors:** Ying-Ping Gai, Ya-Nan Zhao, Huai-Ning Zhao, Chuan-Zhong Yuan, Shuo-Shuo Yuan, Shuo Li, Bing-Sen Zhu, Xian-Ling Ji

**Affiliations:** ^1^State Key Laboratory of Crop Biology, Shandong Agricultural University Tai’an, China; ^2^College of Forestry, Shandong Agricultural University Tai’an, China; ^3^Mountain Tai Forest Ecosystem Research Station of State Forestry Administration Tai’an, China

**Keywords:** mulberry, plant latex, MLX56, tissue expression pattern, defense roles

## Abstract

Biotic stresses are major constraints limiting the leaf quality and productivity of mulberry. MLX56 is a unique chitin-binding protein isolated from Shin-Ichinose (*Morus alba*) latex that displays toxicity against lepidopteran caterpillars. In this study, the full-length cDNA encoding MLX56 was isolated from Husang 32 (*M. multicaulis*) and designated *HMLX56*. Amino acid sequence analysis and protein modeling of three MLX56 proteins showed that they were highly conserved among *Morus* species. Tissue expression pattern analysis showed that the *HMLX56* gene was strongly expressed in mulberry bark and leaves but only slightly expressed in fruits. In addition, analysis of GUS expression indicated that the promoter of *HMLX56* showed higher transcriptional activity along the vascular strands, and its activity can be regulated by various environmental factors. Like the MLX56 protein from *M. alba*, the HMLX56 protein showed toxicity to *Plutella xylostella*. Moreover, when the *HMLX56* gene was ectopically expressed in Arabidopsis, the transgenic plants showed enhanced resistance to aphids, the fungal pathogen *Botrytis cinerea* and the bacterial pathogen *Pseudomonas syringae* pv. tomato DC3000. Our data suggest that the HMLX56 protein has a lectin-like molecular structure consisting of two hevein-like chitin-binding domains which provide not only chitin-binding activities but also other mechanisms of defense. The information provided here improves our understanding of the potential functions and defense mechanisms of MLX56 proteins, enabling in-depth functional analysis of latex exudates and perhaps facilitating mulberry genetic improvement in the future.

## Introduction

Mulberry (*Morus* spp.) is the sole food plant of the silkworm (*Bombyx mori* L.) (Ji et al., 2009; Zhang et al., 2011) and is often affected by a number of diseases and herbivores, which may strongly affect leaf quality and productivity (Kumar and Gupta, 2004). Since mulberry leaves are used to feed silkworms, the improper use of agrochemicals to treat those diseases and herbivores could be hazardous to silkworms (Ji et al., 2008). Therefore, it is thus fundamental to improve mulberry characteristics through the development of mulberry varieties with increased tolerance to diseases and herbivores. During the course of evolution, plants have developed diverse morphological, functional and chemical adaptations to ward off pathogens and pests (Odintsova et al., 2009). Laticifers are specialized, elongated latex-secreting cells that may occur as single cells or a series of interconnected cells (Pickard, 2008). The network of laticifer cells is one of the most important conduit systems in higher plants (Trindade et al., 2006), and it can be found in more than 20,000 species of angiosperm plants from over 40 families (Lewinsohn, 1991). Latex is a sticky sap that flows out of the points of laticiferous tissue upon mechanical wounding or insect herbivory (Konno, 2011) and contains several classes of secondary metabolites and proteins (Agrawal and Konno, 2009; Ramos et al., 2014; Freitas et al., 2015). The best-studied proteins in latex are peptidases, peptidase inhibitors, chitinases, and anti-oxidative enzymes. Recently, it was demonstrated that latex proteins have various biological functions including transcription, translation, protein degradation and response to environmental stimuli (Cho et al., 2014), and some of them play roles in protecting plants against insects and fungi (Looze et al., 2009; Konno, 2011; Ramos et al., 2014; Freitas et al., 2015). However, until recently, it was not clear in most cases whether the defense proteins in latex have roles in activating the precursor molecules of defense chemicals or synthesizing defense chemicals (Konno, 2011). Therefore, the role of latex proteins in plant defense is still a developing area of research, and the mechanisms underlying their protective action are mostly unknown.

In a recently published study on mulberry latex, a novel defense protein, MLX56, was purified from *M. alba* (Shin-Ichinose) latex. The protein showed strong toxicity to generalist lepidopteran herbivores at very low concentrations, but the mulberry specialist silkworm (*B. mori*) was not at all affected (Wasano et al., 2009; Konno, 2011). The two hevein-like chitin-binding domains of the MLX56 protein confer strong chitin-binding activity (Wasano et al., 2009). Chitin-binding proteins (CBPs) are present in many species including plants, and some CBPs can interfere with fungal growth and be toxic to insects by binding to and disrupting the proper function of chitin (Van Damme et al., 1998; Kasprzewska, 2003; Trindade et al., 2006; Manjeet et al., 2013; Batista et al., 2014). Moreover, the MLX56 protein has a chitinase-like domain, but it has no chitinase activity. It remains unclear whether the toxic mechanism of MLX56 resembles that of CBPs. Furthermore, whether the MLX56 protein plays a role in defense against bacterial pathogens remains elusive. Though the plants of the Moraceae family are characterized by the presence of latex (Rahman and Khanom, 2013), latex ingredients are diverse even among species in the same genera (Wasano et al., 2009). The MLX56 protein was found to be unique to mulberry trees (*Morus* species) among Moraceae genera (Wasano et al., 2009; Konno, 2011). However, the conservation of the *MLX56* gene among *Morus* species is currently unclear. Moreover, the expression levels of MLX56 in different organs of mulberry are still undescribed, and the transcriptional regulation mechanism of the gene has not yet been elucidated.

In this study, the *MLX56* gene was cloned from *M. multicaulis* (Husang 32), and the conservation and expression patterns of the gene were analyzed. Meanwhile, the roles of the gene in defense against insects and pathogens were investigated. Moreover, the potential defense mechanisms of MLX56 proteins were discussed. Our collective findings might shed light on the characteristics and functions of *MLX56* genes and will assist greatly in understanding the defense mechanisms of MLX56 protein and provide a foundation to explore potential genes to be used in mulberry biotechnology in the future.

## Materials and Methods

### Biological Materials

*Morus multicaulis* (Husang 32) was incubated in a growth chamber at 26°C, humidity 50–60% and 12 h of light. *Arabidopsis thaliana* (Col-0) plants were grown at 22°C, humidity 50–60% and 12 h of light.

### Cloning and Sequence Analysis

RNA was isolated from leaves of *M. multicaulis* using TRIzol^®^ reagent following the manufacturer’s recommendations (Invitrogen) and digested with DNase I. cDNA was synthesized using 2 μg of total RNA with 100 units of reverse transcriptase M-MLV (Promega) in 20 μL reactions. The specific oligonucleotide primers (MLX56-5′: 5′-GCATGAAGTTTAGAACTCTTCT-3′; MLX56-3′: 5′-TTACATTCGAGCAACTTCCG-3′) were designed based on our available mulberry transcriptome data for PCR amplifications, and the DNA fragments obtained from RT-PCR were subcloned individually into the pMD18-T vector (Invitrogen) resulting in pMD18-MLX56. After transformation, positive clones were selected and further sequenced. The deduced amino acid sequences of *MLX56* genes were aligned using DNAMAN multiple alignments program. Structural prediction was performed with SWISS-MODEL tools^1^.

### Promoter Analysis

To obtain the promoter sequence of *MLX56* from Husang 32, chromosome walking was performed using the TAIL-PCR method. Three specific primers (SP1: 5′-TTGGCAACCTTGATCAACATCACA-3′, SP2: 5′-ACGATGGACTCCAGTCGGCATAAGGCACCTCCTACA-3′ and SP3: 5′-TGTTGCTCACTACAATTTCTAGCAGAA-3′) were designed using the *MLX56* cDNA sequence. Four arbitrary primers were used (LAD-1:5′-ACGATGGACTCCGVNVNNNGGAA-3′; LAD-2: 5′-ACGATGGACTCCAGAGCGBNBNNNGGTT-3′; LAD-3: 5′-ACGATGGACTCCAGAGCGBDNBNNNCGGT-3′; LAD-4: 5′-ACGATGGACTCCAGAGCGHNVNNNCCAC-3′) and the AC1 (5′-ACGATGGACTCCAGAG-3′) primer complementary to an adaptor sequence within the LAD primers was designed according to the previous study (Liu and Chen, 2007). Mulberry genomic DNA was isolated using the cetyltrimethyl-ammonium bromide method as previously described (Sato et al., 1996) and subjected to preamplification using the primers LAD and SP1. The amplification product was diluted and used as the template in the primary TAIL-PCR using the primer pairs AC1 and SP2. By using the same method, the primary TAIL-PCR product was used as a template in the secondary TAIL-PCR using the primers AC1 and SP3. The secondary TAIL-PCR amplified products were analyzed on 1.0% agarose gels, and the bands of interest were isolated and purified using a universal genomic DNA extraction kit (TaKaRa) and ligated to pMD18-T vector, then transformed into *Escherichia coli* for sequencing. The potential *cis*-regulatory elements within the promoter were analyzed using the PlantCARE program online^2^.

The promoter was cloned into the vector pBI121 to replace the cauliflower mosaic virus (CaMV) 35S promoter and fused to the GUS (β-glucuronidase) reporter gene to create the promoter expression vector pHMLX56::GUS, and the derived construct vector was introduced into *Agrobacterium tumefaciens* strain GV3101. For Arabidopsis transformation, the floral dipping method (Clough and Bent, 1998) was employed. Histochemical staining for GUS activity was performed as described by Jefferson (1987).

### Plant Treatment

Two-week-old transgenic and wild-type Arabidopsis seedlings were grown in the growth chamber under conditions specified above. The abscisic acid (ABA), gibberellin (GA) and salicylic acid (SA) treatments were achieved by spraying the rosette leaves with 100 μM ABA, 200 μM GA, or 5 mM SA solution. Control plants were sprayed with water. To test the effects of pathogen stimuli on the activity of the promoter, Arabidopsis seedlings were inoculated with *Pseudomonas syringae* pv. tomato DC3000 (*Pst* DC3000) by spraying *Pst* DC3000 bacterial suspensions (10^5^ CFU mL^-1^) onto the rosette leaves. Water was used as control. All samples from the above treatments were harvested 12 h post-treatment. For wounding treatment, the leaves were squeezed with a tweezer, and the wounded seedlings were harvested 4 h later. For dark and light treatments, seedlings were incubated continuously in the dark or light for 24 h. Each treatment was replicated three times with 10 seedlings per replicate. All the samples described above were immediately frozen in liquid nitrogen after harvest and stored at -80°C for GUS fluorometric assays.

### GUS Activity Assay

To assay GUS activity, Arabidopsis leaves were harvested and stored at -80°C before use. Frozen leaves were ground in extraction buffer (50 mM pH 7 sodium phosphate, 10 mM EDTA, 0.1 Sarkosyl, 0.1 M Triton X-100, and 10 mM β-mercaptoethanol). The homogenate was centrifuged at 12,000 × *g* for 15 min (4°C), then the supernatant was used for GUS activity assays. GUS activity was assayed by using 10 μL extract and 4-methyl-umbelliferyl-glucuronide (Sigma) as a substrate. The protein concentration of the extracts was determined utilizing bovine serum albumin as a standard protein according to the assay described by Bradford (1976). Fluorescence was measured in a fluorescence spectrophotometer (Hitachi, Tokyo, Japan). The excitation wavelength was 365 nm and the emission wavelength 455 nm. Each assay was repeated three times. The data presented were collected from at least three independent experiments.

### Quantitative Real-time PCR Analysis

Total RNAs were extracted from leaves, stems, roots, flowers and fruits of *M. multicaulis* (Husang 32) plants, and cDNA was synthesized as described above. Real-time PCR was performed using the SYBR Premix Ex Taq^TM^ kit (TaKaRa) according to the manufacturer’s protocol on the Rotor-Gene 3000A system. The *EF1-*α gene was amplified as a reference gene for mRNA normalization. The *MLX56* gene was amplified using primer pair F (5′-TGTAATCCAGGAAGGTGTTGTAG-3′) and R(5′-GAGAAGTCCAACATTGGTATTG-3′), and the *EF1*-α gene was amplified using primer pair F (5′-ATGGTGAAGATGATTCCCACTAAGC-3′) and R (5′-AAAAGCCAGTCACTTCCCTCCCT-3′). Comparative cycle threshold (*C*t) method (Livak and Schmittgen, 2001) was used to evaluate the relative gene expression level. All samples were assayed in triplicate.

### Production of Transgenic Plant Lines

The coding region of the *MLX56* gene was amplified from pMD18-MLX56 plasmid DNA with *Xba* I sense primer (5′-TCTAGAATGAAGTTTAGAACTCTT-3′) and *Sac* I antisense primer (5′-GAGCTCTTACATTCGAGCAACT-3′). The PCR-amplified fragment was cloned into pMD18-T plasmid, which was then digested with *Xba* I and *Sac* I. The products were analyzed by 1% agarose gel electrophoresis, and a DNA fragment of approximately 1200 bp was recovered and subcloned into binary plasmid vector pBI121 (digested with *Xba* I and *Sac* I) under the control of the 35S promoter. Then, the vector was introduced into *A. tumefaciens* strain GV3101, and wild-type Arabidopsis plants were transformed with the floral dip method. After transformation, the sterilized T1 seeds were plated on kanamycin selection plates (MS media supplemented with 50 μg mL^-1^ kanamycin) to select transformed plants.

### Northern and Western Blotting

For northern blotting, total RNA was extracted, separated and then blotted onto a nylon Hybond N membrane. The blots were hybridized with digoxigenin-labeled RNA probes prepared using the PCR DIG Probe Synthesis Kit (Roche). Prehybridization, hybridization, membrane washing, and detection were performed according to the method described by Umezawa et al. (2006). For western blotting, protein was prepared and mixed with 5 × SDS-PAGE sample buffer. Then, the samples were heated at 95°C for 3 min and separated by SDS-PAGE on 12% SDS-polyacrylamide gels. After electrophoresis, the separated proteins were electroblotted onto nitrocellulose membranes. Western blot analysis was performed using the anti-*MLX56* protein polyclonal antibody according to a previously described method (Poppenberger et al., 2011).

### Morphological Characterization Analysis of Transgenic Plants

Surface-sterilized Arabidopsis seeds from transgenic Arabidopsis lines expressing *HMLX56* gene and wild-type were sown on MS media and kept in darkness for 2 days at 4°C to synchronize germination. Then the seeds were germinated on MS medium and grown vertically in a growth chamber at 22°C on a 16/8 h light/dark cycle for root morphology examination. At the same time, the seeds were planted in square plastic pots filled with mixed soil (vermiculite: humus = 1:1) and cultured under the same condition. Height, number of rosette leaves and shoots, and days to flowering were measured over a 6-week period. These experiments were carried out with three replications and six plants per replication.

### Insecticidal Activity Assay

*Plutella xylostella tests* The eggs of *P. xylostella* were hatched in an incubator at 26°C, and caterpillars of approximately 1 mg in size were placed on the leaves of 4-week-old Arabidopsis plants with two individuals per plant. The weights of caterpillars were determined 6 days later. This experiment was conducted with three replicates and 10 seedlings per replicate.

*Aphid tests* Stock colonies of *Myzus persicae* were reared on Chinese cabbage (*Brassica rapa*, ssp. chinensis) under standard conditions in a growth incubator at 25°C, 60% relative humidity and 16 h photoperiod. Synchronized 1-day-old nymphs were used to infest 4-week-old Arabidopsis plants with two nymphs per plant. The average weight of aphids on plants was measured at 5 days after introduction, and the total number of nymphs was calculated every 4 days for 16 days after introduction. Three replications of this experiment were carried out with 10 seedlings per replicate.

### Pathogen Inoculation and Disease Resistance Assay

Four-week-old transgenic Arabidopsis and wild-type plants were used for disease resistance analysis. Leaves of each plant were detached with a sharp blade and challenged with *Botrytis cinerea* by applying 2 mm diameter plugs of PDA media containing actively growing mycelia of *B. cinerea*. The leaves were then placed on wet filter paper in a covered culture dish to maintain high humidity, and incubated at 22°C. Disease incidence and lesion sizes, presented as the diameter of the lesion (mm), were surveyed. Inoculation with *Pst* DC3000 was conducted by infiltrating with 50 μL bacterial suspensions (10^5^ CFU mL^-1^) using 1-mL syringes without needles, and the disease symptoms were recorded using a camera. Chlorophyll abundance was measured to assay chlorosis development in the inoculated leaves. Leaf disks from three separate leaves were frozen in liquid nitrogen and then homogenized in 80% (v/v) acetone. The homogenates were centrifuged at 500 × *g* for 3 min at 4°C, and the supernatant was used for chlorophyll assays. The amount of chlorophyll was determined on a scanning spectrophotometer as previously described (Ritchie, 2006). Each treatment was replicated three times with 10 seedlings per replicate.

### Statistical Analysis

Data are reported as means ± SD of at least three independent experiments. Statistical significance was subjected to Duncan’s multiple range test with analysis of variance (ANOVA) using Statistical Analysis System software (v. 9.3 for Window; SAS Institute, Cary, NC, United States, 2010).

## Results

### *MLX56* Genes Are Conserved among *Morus* Species

**FIGURE 1:**
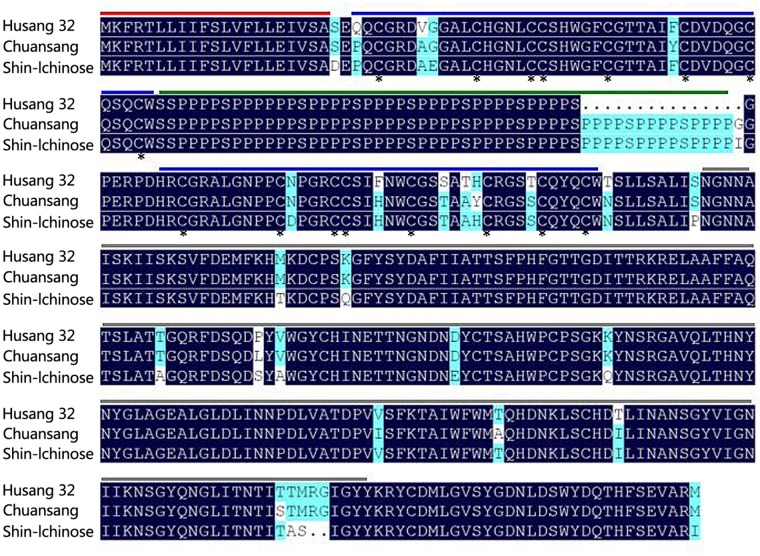
Comparison of the amino acid sequence of the HMLX56 protein (JX432966.1) with the MLX56 proteins from *Morus alba* (Shin-Ichinose) (EF535852.2) and *M. notabilis* (Chuansang) (MF192878). Amino acid residues conserved in all proteins were black shaded and similar amino acids were gray shaded. Signal peptide regions are lined above the sequence in red, putative hevein-like chitin-binding domains in blue, extensin domains in green, and chitinase-like domains in gray. Conserved cysteine residues are marked with asterisks.

**FIGURE 2:**
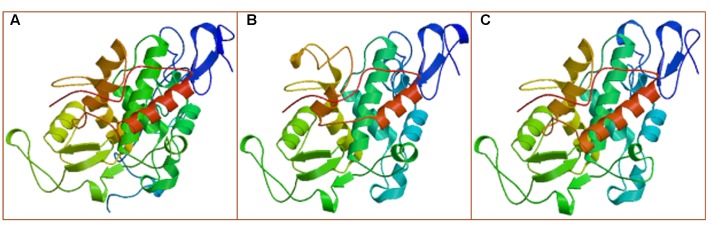
The proposed 3-D structures of the MLX56 proteins established by homology-based modeling. **(A–C)** Are the 3-D structures predicted for MLX56 proteins from *M. multicaulis*, *M. notabilis*, and *M. alba*, respectively.

The *MLX56* gene was isolated from *M. multicaulis* by PCR and designed as *HMLX56* (Bank Accession No. JX432966). The open reading frame (ORF) encoded a protein of 400 AA which shows 91 and 93% amino acid sequence identity with those of *M. notabilis* (Chuansang) and *M. alba* (Shin-Ichinose), respectively (**Figure 1**). The signal peptide (amino acid residues 1–21) predicted by SIGNALP V3.0 in the N-terminal region of HMLX56 protein was identical to those of other MLX56 proteins (**Figure 1**). When the signal peptide was removed, the mature MLX56 proteins of *M. multicaulis*, *M. notabilis*, and *M. alba* had similar structures comprising three domains. The first part, consisting of two hevein-like chitin-binding domains, was well conserved between different *Morus* species, and this domain had eight conserved cysteine residues which were essential for keeping the functional structure by four intrachain disulphide bridges. Though the numbers of Ser[Pro]_n_ repeats in the middle of the two chitin-binding regions were different between HMLX56 and the other two MLX56 proteins, the extensin domain (motif) was well conserved among the three MLX56 proteins. In addition, the third part, consisting of a C-terminal chitinase-like domain, was also well conserved among the three proteins (**Figure 1**). Molecular modeling results indicated that the spatial architecture of the HMLX56 protein is very similar to those of the MLX56 proteins from Chuansang and Shin-Ichinose (**Figure 2**). These data revealed that the MLX56 proteins are conserved among *Morus* species, and they may have similar functions.

### Gene Expression Pattern of *HMLX56*

**FIGURE 3:**
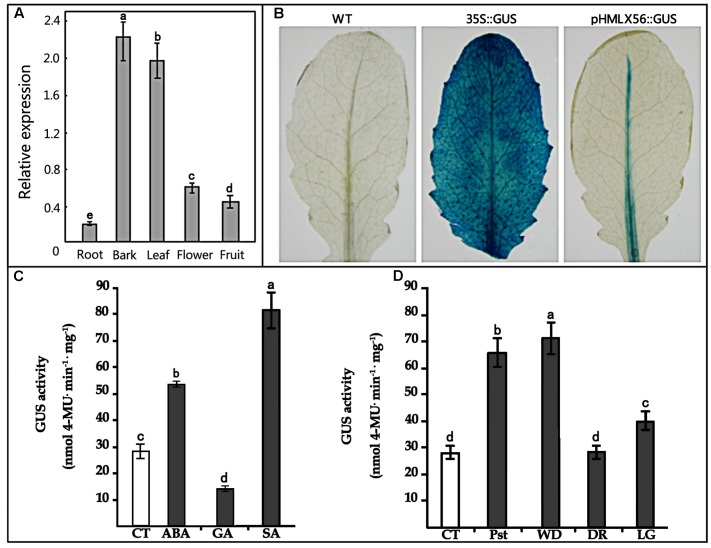
Expression of the *HMLX56* gene in different tissues. **(A)** mRNA expression levels of *HMLX56* in mulberry tissues as analyzed by qRT-PCR. The relative gene expression was evaluated using comparative *C*t method with *EF1-*α as the reference gene. The log2 values of the ratio of the expression of *HMLX56* to *EF1-*α are plotted. Data are the average of three experiments for three test samples. Error bars represent SD. **(B)** Stable expression of pHMLX56::GUS in Arabidopsis plants. The expression vector pHMLX56::GUS was constructed by cloning the promoter of *HMLX56* into the vector pBI121 to replace the cauliflower mosaic virus (CaMV) 35S promoter and drive expression of the GUS (β-glucuronidase) reporter gene. The plasmid pBI121 containing 35S::GUS was used as a positive control, and wild-type Arabidopsis plants were used as negative controls. **(C,D)** GUS activity driven by the *pHMLX56* promoter in transgenic plants as measured by spectrophotometer. Assays were performed three times, each time with three replicates. Values are given as the mean ± SD of three experiments in each group. Different letters above the columns indicate significant differences (*P* < 0.05) according to Duncan’s multiple range test. WT, wild type; CT, control; Pst, *Pst* DC3000 infection; WD, wound treatment; DR, dark treatment; LG, light treatment.

To obtain better insight into the biological function of the *HMLX56* gene, we first investigated its expression levels in different organs of Husang plants. The RT-PCR analysis indicated that the *HMLX56* gene was constitutively expressed in mulberry organs, but its expression level varied considerably across organs (**Figure 3A**). The *HMLX56* gene was highly expressed in leaves and bark, but it was expressed at a low level in flowers and fruits and at the limits of detection in the roots. To explore the precise expression patterns of *HMLX56* at the tissue level, the putative promoter, 1848 bp DNA upstream of the *HMLX56* coding region sequence, was cloned (designated *pHMLX56*), and a plant expression vector containing *pHMLX56* promoter fused to *GUS* gene was constructed and introduced into Arabidopsis plants. These transgenic plants were subjected to histological GUS analysis to investigate the expression location of *HMLX56* gene. Strong GUS signals were detected around the vascular cylinder of leaves indicating that the *HMLX56* gene was a tissue-specific gene and might be expressed specifically around the vascular cylinders (**Figure 3B**). *Cis*-acting regulatory elements analysis of the sequence of *pHMLX56* showed that it contains some *cis*-acting elements involved in the light, ABA, wounding and pathogen responses (**Table 1**). To investigate whether these environmental factors were involved in the regulation of *pHMLX56* activity, the regulatory patterns of *pHMLX56* under treatment with ABA, SA, GA, *Pst* DC3000, wounding, light and dark were analyzed. Using fluorometry, an obvious induction of GUS activity was observed in *pHMLX56* plants upon treatment by SA, ABA, *Pst* DC3000 and wounding, and lower GUS activity was observed in the *pHMLX56* plants upon treatment by light. No change in GUS activity was observed upon dark treatment. In contrast, when exposed to GA, GUS activity was significantly decreased (**Figures 3C,D**). These results indicate that *HMLX56* promoter activity can be regulated by ABA, SA, GA, *Pst* DC3000, wounding, and light, but with different activity levels in response to different environmental factors.

**Table 1:** *Cis*-acting regulatory element analysis of the promoter of *HMLX56* gene.

Number	Site name	Amount	Sequence	Function of site
1	AAGAA_motif	1	GAAAGAA	Found in oats, unknown function
2	ABRE	3	CACGTG or TACGTG or TACGTGTC	*Cis*-acting element involved in abscisic acid responsiveness
3	AT-rich element	1	ATAGAAATCAA	Binding site of AT-rich DNA binding protein (ATBP-1)
4	ACE	1	ACGTGGA	*Cis*-acting element involved in light responsiveness
5	ATCT-motif	1	AATCTAATCC	Part of a conserved DNA module involved in light responsiveness
6	ATGCAAAT-motif	1	ATACAAAT	*Cis*-acting regulatory element associated to the TGAGTCA motif
7	Box 4	4	ATTAAT	Part of a conserved DNA module involved in light responsiveness
8	Box-W1	1	TTGACC	Fungal elicitor responsive element
9	CAAT-BOX	32	CAATT or CAAT or CAAAT or CCAAT	Common *cis*-acting element in promoter and enhancer regions
10	CATT-motif	1	GCATTC	Part of a light responsive element
11	G-BOX	2	CACGTG or CACGTA	*Cis*-acting regulatory element involved in light responsiveness
12	G-box	1	CACGTG or TACGTG	*Cis*-acting regulatory element involved in light responsiveness
13	HSE	3	AAAAATTTC	*Cis*-acting element involved in heat stress responsiveness
14	LAMP-element	1	CTTTATCA	Part of a light responsive element
15	P-box	2	CCTTTTG	Gibberellin-responsive element
16	TATA-BOX	59	TATA	Core promoter element around -30 of transcription start
17	W box	1	TTGACC	Wounding and pathogen responsiveness. Binds WRKY type transcription factors
18	TCA-element	1	CAGAAAGGA	*Cis*-acting element involved in salicylic acid responsiveness
19	TC-rich repeats	1	ATTCTCTAAC	*Cis*-acting element involved in defense and stress responsiveness


### Ectopic Expression of *HMLX56* Gene in Arabidopsis Has No Effect on Plant Growth and Development

**FIGURE 4:**
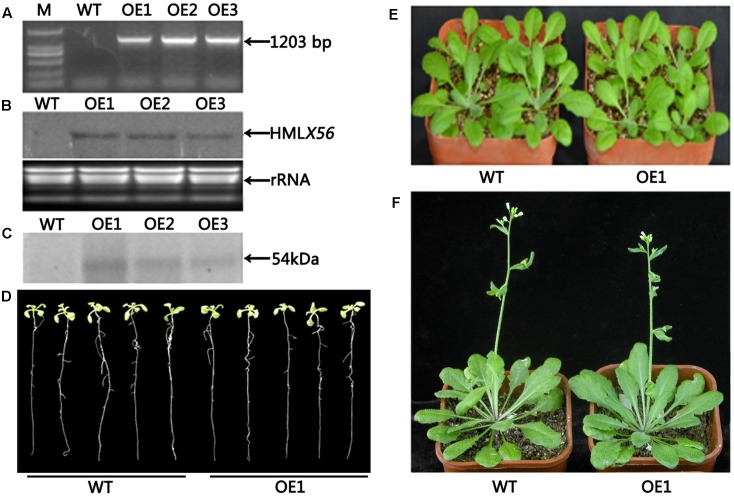
Identification and characterization of the *HMLX56* transgenic Arabidopsis lines. **(A)** Confirmation of transgene integration into the Arabidopsis genome by PCR. **(B)** *HMLX56* expression in transgenic Arabidopsis plants confirmed by northern blot. **(C)** *HMLX56* expression in transgenic Arabidopsis plants confirmed by western blot. **(D)** One-week-old plant phenotypes of wild-type and overexpression Arabidopsis lines. **(E)** Four-week-old plant phenotypes of wild-type and overexpression Arabidopsis lines. **(F)** Six-week-old plant phenotypes of wild-type and overexpression Arabidopsis lines. M, DNA marker; WT, wild-type; OE, transgenic Arabidopsis plants overexpressing *HMLX56*.

Transgenic Arabidopsis plants constitutively expressing the *HMLX56* gene were generated by transforming wild-type Arabidopsis plants with constructs containing the *HMLX56* ORF sequence under the regulation of the 35S promoter. The *HMLX56* gene was successfully integrated into the Arabidopsis genome (**Figure 4A**) and expressed at detectable mRNA and protein levels in the transgenic Arabidopsis plants (**Figures 4B,C**). All the *HMLX56* over-expression lines did not show significantly different root morphology, number of rosette leaves and shoots, and flowering time compared with the wild-type plants (**Figures 4D–F**). This indicated that ectopic expression of *HMLX56* in Arabidopsis plants has no effect on plant growth and development, and the *HMLX56* gene may not be involved in the process of plant development.

### Transgenic Arabidopsis Plants Expressing the *HMLX56* Gene Display Enhanced Resistance to Insect

**FIGURE 5:**
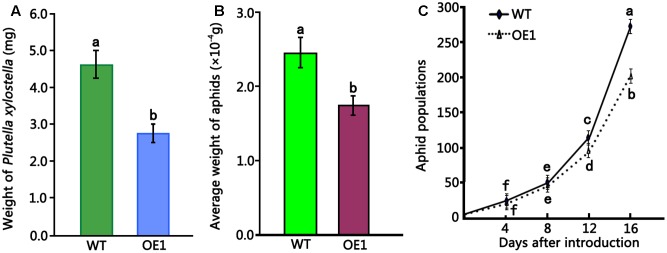
Insect toxicity tests of transgenic Arabidopsis plants overexpressing *HMLX56* against *P. xylostella* larvae and aphids. **(A)** Insect toxicity tests of HMLX56 against the larvae of *Plutella xylostella.* Larval weights were measured at 6 days after introduction. **(B)** Insect toxicity tests of HMLX56 against aphids. Larval weights were measured at 5 days after introduction. **(C)** Aphid populations were counted every 4 days for 16 days after introduction. Bioassays were performed three times, each time with three replicates, and each value is the mean ± SD of three experiments. Different letters above the columns, and those above or under the points indicate significant differences (*P* < 0.05) according to Duncan’s multiple range test. WT, wild-type; OE1, transgenic Arabidopsis plants overexpressing *HMLX56*.

To evaluate the insecticidal activity of HMLX56, 4-week-old wild-type and transgenic (ectopically expressing the *HMLX56* gene) Arabidopsis plants were challenged with *P. xylostella* caterpillars of approximately equal developmental stages and weight, and the weight of caterpillar was determined 6 days later. The results showed that the caterpillars feeding on wild-type Arabidopsis exhibited significantly increased mean weight in comparison to those feeding on transgenic plants (**Figure 5A**). Therefore, the HMLX56 protein exhibits significant growth-inhibitory activity against *P. xylostella* caterpillars. To further investigate the role of HMLX56 protein in insect resistance, we studied the resistance of the *HMLX56*-overexpressing transgenic Arabidopsis to green peach aphid. Two first-instar aphids were transferred to each of the wild-type and transgenic plants grown in the same pot. The average weight of aphids on the plants was measured 5 days later, and the results showed that the average weight of aphids feeding on transgenic plants was significantly lower than that of aphids feeding on the wild-type plants (**Figure 5B**). In addition, the number of aphids was also counted 16 days after introduction, and the population of aphids feeding on the transgenic plants was significantly smaller than that of aphids feeding on the wild-type plants (**Figure 5C**). These data suggested that the expression of *HMLX56* gene in Arabidopsis enhances the resistance of transgenic plants to aphids. Therefore, HMLX56 exhibits significant growth-inhibitory activity against both *P. xylostella* caterpillars and aphids.

### Ectopic Expression of *HMLX56* Gene in Arabidopsis Enhances Resistance to *B. cinerea* and *Pst* DC3000

**FIGURE 6:**
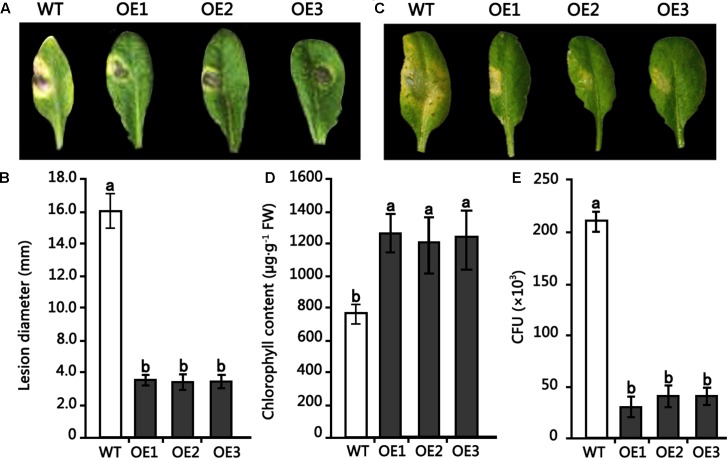
Resistance of transgenic Arabidopsis plants overexpressing *HMLX56* to *Botrytis cinerea* and *Pst* DC3000. **(A)** Symptoms observed 4 DAI on the leaves of 4-week-old Arabidopsis inoculated with 1-mm-diameter plugs of *B. cinerea*. **(B)** Symptoms were quantified at 4 DAI by lesion diameter. **(C)** Disease symptoms in Arabidopsis leaves caused by *Pst* DC3000 infection. **(D)** Chlorophyll amounts in *HMLX56* overexpressors and wild-type Arabidopsis plants after *Pst* DC3000 infection. **(E)** Growth of *Pst* DC3000 strains in inoculated Arabidopsis leaves. The bacterial numbers were calculated at 3 days after inoculation and represented as colony-forming units (CFU) per gram leaf tissue. Each infection assay was performed in triplicate. Each value is the mean ± SD of three replicates. Different letters above the columns indicate significant differences (*P* < 0.05) according to Duncan’s multiple range test. WT, wild-type; OE1-3, transgenic Arabidopsis lines overexpressing *HMLX56*.

To examine the role of the *HMLX56* gene in plant defense response to pathogens, transgenic Arabidopsis plants overexpressing the *HMLX56* gene were inoculated with *B. cinerea* and *Pst* DC3000. When the detached leaves from 4-week-old Arabidopsis plants were inoculated with *B. cinerea*, disease development was analyzed 4 days after inoculation (DAI). The results showed that leaves of wild type plants inoculated yielded expanding disease yellow lesions around the inoculated points. However, the disease lesions around the inoculated points in the inoculated leaves of transgenic plants were smaller compared with those in the wild type plant leaves inoculated (**Figures 6A,B**). To determine whether the *HMLX56* gene is involved in plant response to bacterial pathogen, the plants were inoculated with *Pst* DC3000. Three days after inoculation, the chloroses surrounding inoculation points in the leaves of wild-type plants were larger and more severe than those in the leaves of transgenic plants (**Figures 6C,D**), suggesting that the *HMLX56* gene conferred resistance to *Pst* DC3000 in Arabidopsis. To further confirm this, the bacterial growth in the inoculated leaves was determined, and the results showed that the growth of *Pst* DC3000 strain was extremely limited in the leaves of plants overexpressing *HMLX56* (**Figure 6E**). This was in accordance with the milder symptom development in the plants overexpressing *HMLX56*. Therefore, ectopically expressing the *HMLX56* gene in Arabidopsis enhanced plant resistance to *B. cinerea* and *Pst* DC3000, and the *HMLX56* gene may have roles in the defense response to fungal and bacterial pathogens.

## Discussion

In our study, it was shown by *in planta* experiments that transgenic plants overexpressing HMLX56 exhibited significant growth inhibition of *P. xylostella* caterpillars and aphids and disease resistance to inoculation with *B. cinerea* (**Figures 5**, **6**). Protein structure analysis showed that the N-terminal moiety of HMLX56 has two hevein-like chitin-binding domains (**Figures 1**, **2**) which resemble those of hevein and hevein-like proteins. Hevein and hevein-like proteins possess a characteristic cysteine/glycine-rich putative chitin-binding domain which gives them the ability to bind to chitin, the building block of fungal cell walls and arthropod exoskeletons (Rinaudo, 2006). Hevein-type peptides exhibit various degrees of antimicrobial activity, and it is hypothesized that these proteins can penetrate the fungal cell and affect chitin-containing components, as well as being involved in plant defense against microorganisms and pests (Koo et al., 1998). Since the *MLX56* gene was highly conserved between *M. multicaulis* and *M. alba*, and the MLX56 protein from *M. alba* has been shown to have strong chitin-binding activity (Wasano et al., 2009), HMLX56 may also have strong chitin-binding activity which render it useful in defense. However, even bacteria and fungi containing little or no chitin in their cell wall were strongly inhibited by some hevein-like proteins in previous studies (Koo et al., 1998; Huang et al., 2002; Lee et al., 2003; Nawrot et al., 2014). Here, we also showed that transgenic plants overexpressing *HMLX56* had increased disease resistance to *Pst* DC3000 (**Figure 6**). It has been suggested that chitin in the fungal cell wall is not essential for hevein-like protein defense activities, and the antifungal and growth-inhibitory activities of hevein-like proteins could be mediated by glycoproteins in cell walls instead of chitin (Koo et al., 2002). Moreover, it has been reported that some hevein-like proteins are attracted electrostatically to charged molecules on the membranes of microorganisms through specific cell-surface receptors and can activate several pathways that cause cell death (Pelegrini et al., 2011). Therefore, the hevein-like chitin-binding domains of HMLX56 may provide not only chitin-binding activities but also other mechanisms of defense. In addition to the two hevein-like chitin-binding domains, the C-terminal moiety of MLX56 consists of a chitinase-like domain found in some defense proteins belonging to the family of lectins or chitinases, but MLX56 does not exhibit chitinase activity, nor does the extensin domain of MLX56 have a known function (Wasano et al., 2009). Therefore, the chitinase-like and extensin domains of HMLX56 protein might not have roles in plant defense. Future studies will be carried out to explore which of the three MLX56 domains is responsible for inhibitory activity using transgenic lines including mutant versions of HMLX56.

Plants developed different mechanisms to adapt to challenging environments while facing biotic and abiotic stresses (Rejeb et al., 2014). In the case of plants producing latex, mechanical wounding leads to an abrupt release of latex which will rapidly coagulate, sealing the wound and preventing further entry of pathogens, which may constitute a very efficient defense mechanism (Azarkan et al., 2004). It has been suggested that under oxidative conditions, *Cucurbita* phloem lectins such as PP1 and PP2 are covalently cross-linked via disulphide bonds to form filaments which can be a potent physical barrier against further herbivory and reduce the risk of wound infection by opportunistic pathogens (McCloud et al., 1995; Golecki et al., 1999). Our results indicated that wounding and exogenous application of the stress-related hormone ABA and SA may increase the expression level of *HMLX5*6 (**Figure 3**). Since the HMLX56 protein has a lectin-like molecular structure and disulphide bonds, it might also be cross-linked to form filaments to prevent microbial infection and insect herbivory. Though aphids can use their stylets to deliver salivary chemicals and/or proteins into the sieve elements to prevent or reverse sieve element occlusion and avert substantial wounding-related responses from the plant and drink large volumes of phloem sap (Walling, 2008), MLX56 protein in the phloem sap is highly resistant to protease digestion, and has a strong chitin-binding activity, and shows highly toxic to many caterpillars at very low concentrations (Wasano et al., 2009). In spite of the HMLX56 protein is not present in large quantity within the phloem sap, it may impact aphid physiology leading to impairment of aphid growth, development and reproduction, and this was confirmed by our data (**Figures 5B,C**). Interestingly, the mulberry specialist silkworm (*B. mori*) was not at all affected by MLX56 protein (Wasano et al., 2009; Konno, 2011), suggesting that silkworm has developed adaptation to the mulberry defense. Our results indicated that the MLX56 proteins play key roles in mulberry–insect interactions and it can be considered as a potential target for mulberry genetic improvement in the future.

## Conclusion

The data in this study suggest that MLX56 is unique to mulberry trees and is highly conserved among the *Morus* species. In addition, the MLX56 protein has defensive roles against pathogens and herbivorous insects. These results collectively suggest that MLX56, with broad and potent defensive activity, represent novel candidate genes for developing transgenic plants with enhanced resistance to a wide range of phytopathogens.

## Author Contributions

Y-PG conceived the project, designed the experiments and drafted the manuscript. Y-NZ, H-NZ, C-ZY, S-SY, SL, and B-SZ carried out all the experiments and data analysis. X-LJ supervised the analysis and critically revised the manuscript. All authors read and approved the final manuscript.

## Conflict of Interest Statement

The authors declare that the research was conducted in the absence of any commercial or financial relationships that could be construed as a potential conflict of interest.

## Footnotes

1 https://swissmodel.expasy.org/

2 http://bioinformatics.psb.ugent.be/webtools/plantcare/html/

## References

[B1] Agrawal A. A. Konno K. (2009). Latex: a model for understanding mechanisms, ecology, and evolution of plant defense against herbivory. *Annu. Rev. Ecol. Evol. Syst.* 40 311–331. 10.1146/annurev.ecolsys.110308.120307

[B2] Azarkan M. Wintjens R. Looze Y. Baeyens-Volant D. (2004). Detection of three wound-induced proteins in papaya latex. *Phytochemistry* 65 525–534. 10.1016/j.phytochem.2003.12.006 15003415

[B3] Batista A. B. Oliveira J. T. Gifoni J. M. Pereira M. L. Almeida M. G. Gomes V. M. (2014). New insights into the structure and mode of action of Mo-CBP3, an antifungal chitin-binding protein of *Moringa oleifera* seeds. *PLoS ONE* 27:e111427 10.1371/journal.pone.0111427 PMC4210214 25347074

[B4] Bradford M. M. (1976). A rapid and sensitive method for the quantitation of microgram quantities of protein utilizing the principle of protein-dye binding. *Anal. Biochem.* 72 248–254. 10.1016/0003-2697(76)90527-3 942051

[B5] Cho W. K. Jo Y. Chu H. Park S. H. Kim K. H. (2014). Integration of latex protein sequence data provides comprehensive functional overview of latex proteins. *Mol. Biol. Rep.* 41 1469–1481. 10.1007/s11033-013-2992-6 24395295

[B6] Clough S. J. Bent A. F. (1998). Floral dip: a simplified method for *Agrobacterium*-mediated transformation of *Arabidopsis thaliana*. *Plant J.* 16 735–743. 10.1046/j.1365-313x.1998.00343.x 10069079

[B7] Freitas C. D. Silva M. Z. Bruno-Moreno F. Monteiro-Moreira A. C. Moreira R. A. Ramos M. V. (2015). New constitutive latex osmotin-like proteins lacking antifungal activity. *Plant Physiol. Biochem.* 96 45–52. 10.1016/j.plaphy.2015.07.012 26231325

[B8] Golecki B. Schulz A. Thompson G. A. (1999). Translocation of structural P proteins in the phloem. *Plant Cell* 11 127–140. 10.1105/tpc.11.1.127 9878637 PMC144095

[B9] Huang R. H. Xiang Y. Liu X. Z. Zhang Y. Hu Z. Wang D. C. (2002). Two novel antifungal peptides distinct with a five-disulfide motif from the bark of *Eucommia ulmoides* Oliv. *FEBS Lett.* 521 87–90. 10.1016/S0014-5793(02)02829-6 12067732

[B10] Jefferson R. A. (1987). Assaying chimeric genes in plants: the GUS gene fusion system. *Plant Mol. Biol. Rep.* 5 387–405. 10.1007/BF02667740

[B11] Ji X. Lu G. Gai Y. Zheng C. Mu Z. (2008). Biological control against bacterial wilt and colonization of mulberry by an endophytic *Bacillus subtilis* strain. *FEMS Microbiol. Ecol.* 65 565–573. 10.1111/j.1574-6941.2008.00543.x 18631174

[B12] Ji X. L. Gai Y. P. Zheng C. C. Mu Z. M. (2009). Comparative proteomic analysis provides new insights into mulberry dwarf responses in mulberry (*Morus alba* L.). *Proteomics* 9 5328–5339. 10.1002/pmic.200900012 19834890

[B13] Kasprzewska A. (2003). Plant chitinases-regulation and function. *Cell. Mol. Biol. Lett.* 8 809–824. 12949620

[B14] Konno K. (2011). Plant latex and other exudates as plant defense systems: roles of various defense chemicals and proteins contained therein. *Phytochemistry* 72 1510–1530. 10.1016/j.phytochem.2011.02.016 21450319

[B15] Koo J. C. Chun H. J. Park H. C. Kim M. C. Koo Y. D. Koo S. C. (2002). Over-expression of a seed specific hevein-like antimicrobial peptide from *Pharbitis nil* enhances resistance to a fungal pathogen in transgenic tobacco plants. *Plant Mol. Biol.* 50 441–452. 10.1023/A:1019864222515 12369620

[B16] Koo J. C. Lee S. Y. Chun H. J. Cheong Y. H. Choi J. S. Kawabata S. (1998). Two hevein homologs isolated from the seed of *Pharbitis nil* L exhibit potent antifungal activity. *Biochim. Biophys. Acta* 1382 80–90. 10.1016/S0167-4838(97)00148-9 9507071

[B17] Kumar V. Gupta V. P. (2004). Scanning electron microscopy on the perithecial development of *Phyllactinia corylea* on mulberry-II. Sexual stage. *J. Phytopathol.* 152 169–173. 10.1111/j.1439-0434.2004.00821.x

[B18] Lee O. S. Lee B. Park N. Koo J. C. Kim Y. H. Prasad D. T. (2003). Pn-AMPs, the hevein-like proteins from *Pharbitis nil* confers disease resistance against phytopathogenic fungi in tomato, *Lycopersicum esculentum*. *Phytochemistry* 62 1073–1079. 10.1016/S0031-9422(02)00668-4 12591259

[B19] Lewinsohn T. M. (1991). The geographical distribution of plant latex. *Chemoecology* 2 64–68. 10.1007/BF01240668

[B20] Liu Y. G. Chen Y. (2007). High-efficiency thermal asymmetric interlaced PCR for amplification of unknown flanking sequences. *Biotechniques* 43 649–650, 652 654 passim. 10.2144/000112601 18072594

[B21] Livak K. J. Schmittgen T. D. (2001). Analysis of relative gene expression data using real-time quantitative PCR and the 2^-ΔΔ^C_T_ method. *Methods* 25 402–408. 10.1006/meth.2001.1262 11846609

[B22] Looze Y. Boussard P. Huet J. Vandenbussche G. Raussens V. Wintjens R. (2009). Purification and characterization of a wound-inducible thaumatin-like protein from the latex of *Carica papaya*. *Phytochemistry* 70 970–978. 10.1016/j.phytochem.2009.05.005 19527911

[B23] Manjeet K. Purushotham P. Neeraja C. Podile A. R. (2013). Bacterial chitin binding proteins show differential substrate binding and synergy with chitinases. *Microbiol. Res.* 168 461–468. 10.1016/j.micres.2013.01.006 23480960

[B24] McCloud E. S. Tallamy D. W. Halaweish F. T. (1995). Squash beetle trenching behaviour: Avoidance of cucurbitacin induction or mucilaginous plant sap? *Ecol. Entomol.* 20 51–59. 10.1111/j.1365-2311.1995.tb00428.x

[B25] Nawrot R. Barylski J. Nowicki G. Broniarczyk J. Buchwald W. Goździcka-Józefiak A. (2014). Plant antimicrobial peptides. *Folia Microbiol.* 59 181–196. 10.1007/s12223-013-0280-4 24092498 PMC3971460

[B26] Odintsova T. I. Vassilevski A. A. Slavokhotova A. A. Musolyamov A. K. Finkina E. I. Khadeeva N. V. (2009). A novel antifungal hevein-type peptide from *Triticum kiharae* seeds with a unique 10-cysteine motif. *FEBS J.* 276 4266–4275. 10.1111/j.1742-4658.2009.07135.x 19583772

[B27] Pelegrini P. B. Sarto R. P. D. Silva O. N. Franco O. L. Grossi-de-Sa M. F. (2011). Antibacterial peptides from plants: what they are and how they probably work. *Biochem. Res. Int.* 2011:250349 10.1155/2011/250349 PMC3049328 21403856

[B28] Pickard W. F. (2008). Laticifers and secretory ducts: two other tube systems in plants. *New Phytol.* 177 877–888. 10.1111/j.1469-8137.2007.02323.x 18086227

[B29] Poppenberger B. Rozhon W. Khan M. Husar S. Adam G. Luschnig C. (2011). CESTA, a positive regulator of brassinosteroid biosynthesis. *EMBO J.* 30 1149–1161. 10.1038/emboj.2011.35 21336258 PMC3061039

[B30] Rahman A. H. M. M. Khanom A. (2013). A taxonomic and ethno-medicinal study of species from Moraceae (mulberry) family in Bangladesh Flora. *Res. Plant Sci.* 1 53–57.

[B31] Ramos M. V. Souza D. P. Gomes M. T. R. Freitas C. D. T. Carvalho C. P. S. Júnior P. A. (2014). A phytopathogenic cysteine peptidase from latex of wild rubber vine *Cryptostegia grandiflora*. *Protein J.* 33 199–209. 10.1007/s10930-014-9551-4 24596120

[B32] Rejeb I. B. Pastor V. Mauch-Mani B. (2014). Plant responses to simultaneous biotic and abiotic stress: molecular mechanisms. *Plants* 3 458–475. 10.3390/plants3040458 27135514 PMC4844285

[B33] Rinaudo M. (2006). Chitin and chitosan: properties and applications. *Prog Polym. Sci.* 31 603–632. 10.1016/j.progpolymsci.2006.06.001

[B34] Ritchie R. (2006). Consistent sets of spectrophotometric chlorophyll equations for acetone, methanol and ethanol solvents. *Photosynth. Res.* 89 27–41. 10.1007/s11120-006-9065-9 16763878

[B35] Sato M. Mitsuhashi W. Watanabe K. Kawakita H. (1996). PCR detection of mulberry dwarf disease-phytoplasmas in mulberry tissues, phloem sap collected by laser stylectomy and insect vector *Hishimonus sellatus*. *J. Seric. Sci.* 65 352–358. 10.1126/science.273.5273.352

[B36] Trindade M. B. Lopes J. L. Soares-Costa A. Monteiro-Moreira A. C. Moreira R. A. Oliva M. L. (2006). Structural characterization of novel chitin-binding lectins from the genus *Artocarpus* and their antifungal activity. *Biochim. Biophys. Acta* 1764 146–152. 10.1016/j.bbapap.2005.09.011 16257591

[B37] Umezawa T. Okamoto M. Kushiro T. Nambara E. Oono Y. Seki M. (2006). CYP707A3, a major ABA 8′-hydroxylase involved in dehydration and rehydration response in *Arabidopsis thaliana*. *Plant J.* 46 71–82. 10.1111/j.1365-313X.2006.02683.x 16623881

[B38] Van Damme E. J. M. Peumans W. J. Barre A. Rougé P. (1998). Plant lectins: a composite of several distinct families of structurally and evolutionary related proteins with diverse biological roles. *Crit. Rev. Plant Sci.* 17 575–692. 10.1080/07352689891304276

[B39] Walling L. L. (2008). Avoiding effective defenses: strategies employed by phloem-feeding insects. *Plant Physiol.* 146 859–866. 10.1104/pp.107.113142 18316641 PMC2259051

[B40] Wasano N. Konno K. Nakamura M. Hirayama C. Hattori M. Tateishi K. (2009). A unique latex protein, MLX56, defends mulberry trees from insects. *Phytochemistry* 70 880–888. 10.1016/j.phytochem.2009.04.014 19476960

[B41] Zhang S. D. Soltis D. E. Yang Y. Li D. Z. Yi T. S. (2011). Multi-gene analysis provides a well-supported phylogeny of Rosales. *Mol. Phylogenet. Evol.* 60 21–28. 10.1016/j.ympev.2011.04.008 21540119

